# Bilateral Pulmonary Emboli and Deep Venous Thrombi in Association With Chronic Inflammatory Demyelinating Polyneuropathy

**DOI:** 10.7759/cureus.14802

**Published:** 2021-05-02

**Authors:** Christine M Doherty, Gustavo A Heresi, Adriano R Tonelli, Alice Goyanes, Robert Wilson

**Affiliations:** 1 Department of Neurology, Cleveland Clinic Lerner College of Medicine of Case Western Reserve University, Cleveland, USA; 2 Department of Pulmonary Medicine, Cleveland Clinic, Cleveland, USA; 3 Department of Neurology, Cleveland Clinic, Cleveland, USA

**Keywords:** autonomic disturbance, autonomic, neuromuscular, autoimmune neuromuscular disease, chronic inflammatory demyelinating neuropathy, thrombophilia, peripheral neuropathology, preload failure, right heart cath, venous thromboembolism

## Abstract

Chronic inflammatory demyelinating polyneuropathy (CIDP) is an autoimmune neurological disorder primarily affecting the peripheral nervous system. It is important to recognize because treatment with immunomodulators can improve symptoms. We present the case of a 61-year-old man who developed a saddle pulmonary embolus as well as right-sided deep venous thrombi following left knee arthroplasty. Two months later, he had persistent pre-syncopal symptoms with exertion and had developed paraesthesias in both feet. Invasive cardiopulmonary exercise testing revealed preload failure with no evidence of pulmonary hypertension. He was then referred to neurology where clinical history, physical examination, autonomic battery, and nerve biopsy were consistent with a diagnosis of CIDP with autonomic dysfunction. Extensive venous thromboembolism may be a unique presentation of CIDP. The mechanisms, which may lead to hypercoagulability in CIDP, include the presence of systemic inflammation and denervation of peripheral vasculature leading to stasis.

## Introduction

Chronic inflammatory demyelinating polyneuropathy (CIDP) is a rare autoimmune neurological disorder, affecting 1 in 200,000. It classically presents as a symmetric motor neuropathy causing proximal and distal muscle weakness. However, up to 50% of patients can have atypical presentations, including asymmetric sensorimotor, primary sensory, distal and sensory predominant, and sensory polyradiculopathy [1]. The disease can take a relapsing-remitting or progressive course [2]. Abnormalities on cardiovagal and sudomotor autonomic testing are present in 20%-30% of CIDP patients; however, they tend to be mild [3]. Although CIDP is rare it is important to recognize as treatment with immunomodulators can improve symptoms [1].

On a pathological level, CIDP is characterized by an aberrant immune response against peripheral nerves and nerve roots. Nerve biopsies frequently show lymphocytic infiltration of affected nerves and areas of segmental demyelination and remyelination. Both environmental and genetic factors have been implicated in the development of CIDP. Several different mechanisms have been proposed regarding how CIDP may develop following an immune stimulus such as an infection. There may be cross-reactivity between the epitopes of the organism causing the infection and self-antigens (i.e. molecular mimicry). If a pathogen cannot be totally eradicated it may trigger an excessive immune response leading to tissue damage and the subsequent presentation of self-antigens via antigen-presenting cells, leading to epitope spreading. Infection can also activate a non-specific immune response which can further contribute to tissue damage and self-antigen presentation. In terms of genetic factors, genome-wide association studies have identified polymorphisms in several HLA genes and in genes involved in the immune response to be overrepresented in CIDP. CIDP has been reported to co-occur with many other autoimmune disorders. Patients with CIDP have also been found to have altered activity of T cell subsets in the blood and CSF and altered cytokine production. A subset of CIDP patients has even been demonstrated to possess autoantibodies against myelin antigens. All of this evidence points to CIDP being an autoimmune disorder [4,5]. Research has linked numerous other autoimmune disorders to an increased risk for venous thromboembolism (VTE) [6-8].

The risk of VTE in CIDP patients has not been previously studied. However, there have been multiple reported cases of CIDP associated with membranous glomerulonephritis (MGN) [9]. The association of CIDP with an immune-mediated renal disease indicates that there may be more systemic inflammation outside of the peripheral nervous system than previously recognized. The aim of this study was to document a case of atypical CIDP with autonomic dysfunction co-occurring with extensive VTE, to prompt further investigation of VTE risk specifically in CIDP patients.

## Case presentation

**Figure 1:**
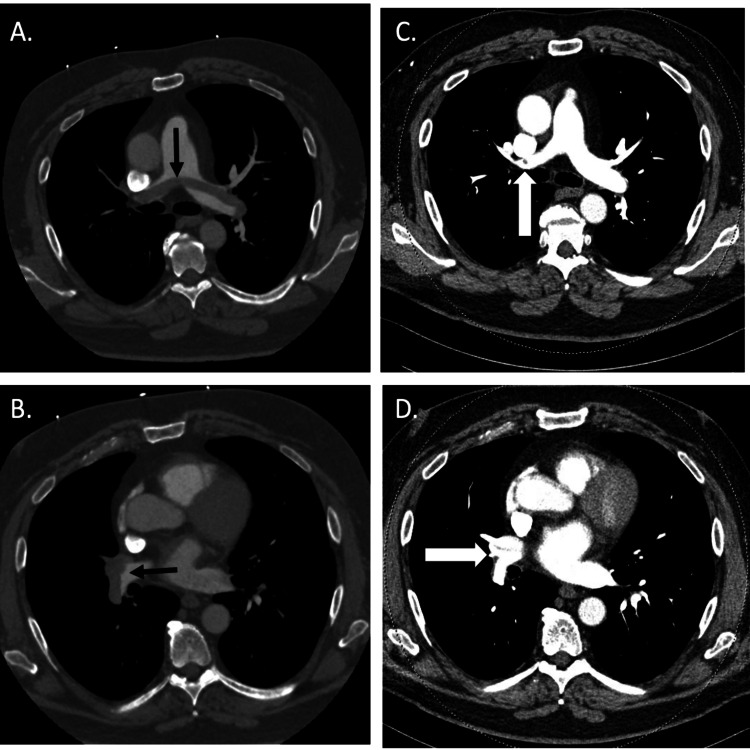
Patient's CT pulmonary angiography Representative images of initial CT pulmonary angiography (left panels) demonstrating acute pulmonary emboli (black arrows) with saddle pulmonary emboli extending into the right upper lobe (A) and right lower lobe (B) pulmonary arteries compared with three months later (right panels) demonstrating residual chronic thrombus (white arrows) at take-off of right upper lobe (C) and right lower lobe (D).

A 61-year-old Caucasian man with a past medical history of hyperlipidemia, obesity (BMI 37 kg/m^2^), and osteoarthritis underwent a left knee arthroplasty (LKA) without complication. After undergoing surgery, he received DVT prophylaxis with two days of low molecular weight heparin while hospitalized and was then discharged home on 81 mg of aspirin daily. Prior to surgery, the patient led an active lifestyle, including regular elliptical use and golfing. Following his LKA he attended all physical therapy sessions. One month following surgery, he abruptly became lightheaded and dyspneic, with no relief of symptoms upon rest. He presented to his local emergency department where he was noted to have sinus tachycardia and hypoxia. CT pulmonary angiogram revealed a large saddle pulmonary embolism with an extension of the clot into multiple lobar and segmental branches (Figures 1A-1D).

Cardiac enzymes were normal and EKG showed no ischemic changes. NT-pro-BNP was elevated at 154 pg/mL. Transthoracic echocardiogram showed moderately increased right ventricular size with moderately depressed right ventricular systolic function, and mild pulmonary hypertension, consistent with the diagnosis of submassive pulmonary embolism. He received high-flow nasal cannula and heparin and was admitted to the ICU.

During his hospital stay, he also had a Doppler ultrasound of the lower extremities revealing a non-occlusive deep venous thrombosis (DVT) in the right popliteal and peroneal veins and a head CT due to dizziness which showed no acute process. He remained hemodynamically stable and supplemental oxygen was weaned. Intravenous anticoagulation therapy with heparin was transitioned to apixaban. He was discharged home on day 6.

The patient presented to our center’s chronic thromboembolic pulmonary hypertension (CTEPH) clinic approximately three and a half months after his submassive pulmonary embolism because of persistent symptoms of postural dizziness and chest pain. He also reported paraesthesias along the back of his head, legs, and feet. His ongoing symptoms were limiting his participation in these activities, and he was unable to return to work as a hospital food service director.

He had no history of prior pulmonary emboli or deep venous thrombi and no known risk factors for VTE, other than recent surgery. Although knee arthroplasty is a known risk factor for VTE, the patient received anticoagulant prophylaxis and remained active post-operatively engaging in frequent physical therapy sessions. Furthermore, the patient developed a DVT in the contralateral leg, making it unlikely that recent orthopedic surgery alone accounted for his significant VTE burden. His risk factors for pulmonary hypertension (all types) included obesity (a risk factor for heart failure with preserved ejection fraction), recreational methamphetamine use 40 years prior to presentation, and anorexigen use for six months 20 years prior to presentation. Surgical history was notable for left knee and hip replacements. His family history was notable for one brother who had a pulmonary embolism. He denied current tobacco or drug use and drank one glass of wine per day. The physical exam at this time was normal.

His chronic thromboembolic pulmonary disease evaluation included blood work (largely unremarkable - including negative anticardiolipin and beta-2-glycoprotein antibodies), imaging and pulmonary and hemodynamic testing. Ventilation-perfusion scan demonstrated no defects, though CT pulmonary angiogram showed chronic thromboemboli in multiple lobar arteries. Transthoracic echo was unremarkable, including normal right ventricular size and function. Pulmonary function tests and a six-minute walk test were also normal.

He underwent an invasive cardiopulmonary exercise test (iCPET) to understand the hemodynamic significance of his chronic thromboemboli. Baseline/resting right heart catheterization revealed mild post-capillary pulmonary hypertension (mean pulmonary artery pressure of 29 mmHg, pulmonary capillary wedge pressure of 18 mmHg) with preserved cardiac index (2.99 L/min/m^2^). With exercise, he developed a pattern consistent with preload failure caused by inadequate cardiac venous return that limits the normal stroke volume increase during activities [10]. To investigate the preload failure noted on iCPET, he was tested for adrenal insufficiency and small fiber neuropathy. ACTH stimulation testing was normal. Skin biopsy showed evidence of distal small fiber sensory neuropathy.

He was then referred for evaluation by our neuro-autonomic dysfunction center. The patient reported no change in symptoms. He remained active and continued exercising using a recumbent bike several times weekly. Neurological examination showed intact sensation to light touch, pinprick, and temperature, normal position sense, normal finger vibratory sense, but markedly reduced toe vibratory sense. Deep tendon reflexes were 2/4 biceps, 1/4 brachioradialis, 1/4 patellar, and 0/4 achilles bilaterally. Toes were silent bilaterally. No coordination deficits were seen. The patient had a sensory ataxic gait, and could not tandem. Romberg sign was present. He had normal muscular strength throughout and no cranial nerve deficits. History, exam, and skin biopsy results were concerning for large and small-fiber sensory neuropathy with autonomic dysfunction. The patient underwent further laboratory screening including erythrocyte sedimentation rate, an autoimmune panel, vitamin B12 level, serum and urine protein electrophoresis, serum-free light chain assay, ganglionic acetylcholine receptor antibodies, and onconeuronal antibodies; which were all unrevealing. QSART and tilt table tests were normal. However, autonomic reflex battery revealed reduced heart rate variability and a low expiratory to inspiratory (E:I) ratio consistent with cardiovagal dysfunction.

**Figure 2:**
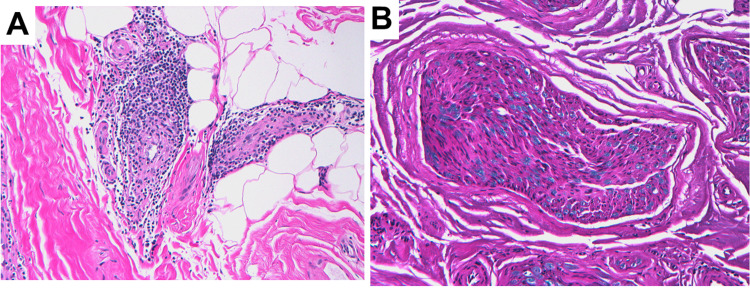
Biopsy of the right sural nerve (A) Epineurial blood vessels marked by a perivascular chronic inflammatory cell infiltrate and focal infiltration of the vessel wall by lymphocytes (hematoxylin and eosin, original magnification 200x). (B) Nerve fascicle marked by a mild loss of large myelinated axons (Luxol fast blue, original magnification 200x).

Electromyography and lumbar puncture could not be safely performed due to the patient’s need for continuous anticoagulation. A right sural nerve biopsy was performed to further investigate the etiology of the sensory neuropathy. It showed perivascular chronic inflammation with non-necrotizing vasculitis (Figure 2A) and mild loss of myelinated axons (Figure 2B), consistent with CIDP. The patient received intravenous immunoglobulin (IVIG) with resulting in improvement in his sensory ataxic gait and a reduction in his autonomic symptoms.

## Discussion

CIDP is a chronic immune-mediated neurological disease. It can affect patients of all ages, but the peak incidence is among patients aged 50-70. Approximately two-third of patients will have a continuously progressive form of disease, while the other third will have a relapsing-remitting course. One study found that six years after disease onset, 56% had a good outcome, 24% no longer responded to treatment, and 11% died due to disease complications. There are three front-line treatments, corticosteroids, plasmapheresis, and IVIG. 50%-70% of patients will respond to their first treatment. However, increasingly in clinical practice, IVIG is being used as a first-line treatment, due to the preferable side effect profile. Indeed, a randomized control trial comparing IVIG to IV methylprednisolone showed that IVIG was more effective and better tolerated than steroids during the first six-month of treatment. Corticosteroids have a plethora of long-term side effects most notably vertebral compression fractures, obesity, diabetes, hypertension, and cataracts. Plasmapheresis requires secure vascular access, and the placement of a central venous catheter can be complicated by pneumothorax, hematoma, brachial plexus injury, and serious infection. Adverse reactions to IVIG tend to be minor (headache, myalgias, nausea, rash) and occur in less than 10% of patients. Thrombotic events such a stroke, MI, retinal vein occlusion, and DVT can also occur. Risk factors for thrombotic events include cardiovascular risk factors, increased serum viscosity, and infusion rates >0.4 g/kg/day [1]. Current FDA guidelines for IVIG recommend ensuring adequate hydration and careful monitoring of patients deemed at high risk for thromboembolism. A recent study found that among patients receiving IVIG specifically for inflammatory neuropathies there was a greater incidence of arterial and venous thromboembolic events compared to population-based rates obtained from hospital admission data. However, this study was comparing rates of thromboembolism among patients with inflammatory neuropathy versus patients hospitalized in the UK for any reason. Of note, 24% of the patients with inflammatory neuropathies required mobility aids. Therefore, it is impossible to know whether their increased embolic risk was due to their inflammatory disease, immobility, or the IVIG itself. The authors note that further study is required to determine whether the current primary prevention guidelines for IVIG treatment are appropriate for patients with inflammatory neuropathies [11].

CIDP is characterized by an aberrant immune response against the peripheral nervous system, which may occur due to molecular mimicry secondary to an immune stimulus or epitope spreading secondary to an overactive immune response. Evidence of CIDP being an autoimmune disorder includes overrepresentation of polymorphisms in genes involved in the immune response among patients with CIDP, the co-occurrence of CIDP with other autoimmune disorders, and the presence of autoantibodies to myelin antigens in a subset of CIDP patients [4,5].

CIDP was not included in either the Swedish or English cohorts, and the risk of VTE in CIDP patients has not been well studied. However, there have been multiple reported cases of CIDP associated with MGN [10]. MGN is an immune-mediated disease thought to be caused either by deposition of antigen-antibody complexes on the glomerular basement membrane or the formation of an antibody that has reactivity against a glomerular antigen [9]. The association of CIDP with an immune-mediated renal disease indicates that there may be more systemic inflammation outside of the peripheral nervous system than previously recognized. Furthermore, the increased rate of embolic events observed in patients with inflammatory neuropathies (including CIDP) treated with IVIG compared to the general hospitalized population may be due at least in part to the underlying inflammatory disorder itself [11].

Virchow’s triad defines the three major factors which lead to VTE as stasis, changes in blood coagulability, and alterations in the vessel wall [9]. Systemic inflammation could have induced hypercoagulability in this patient. Another possible mechanism for the high clot burden in our patient is increased stasis due to denervation of the peripheral vasculature. Our patient exhibited preload insufficiency and had evidence of cardiovagal dysfunction. This indicates that the patient likely has damaged autonomic fibers leading to impaired venoconstriction and resulting venous stasis.

Prior to being diagnosed with CIDP, the patient was diagnosed with preload failure. Preload failure is characterized by an impairment in venous return to the heart, a limitation that becomes clinically relevant when performing activities in the upright position, given the increase in venous pooling with gravity. A reduced venous return can lead to inadequate stroke volume causing symptoms such as dyspnea as well as a postural dizziness-the predominant symptom in our patient. Dyspnea may be explained by different mechanisms, but an increase in the pulmonary ventilation/perfusion mismatch, due to lower flow in the lung apices, is the predominant hypothesis. Preload failure has not previously been described in conjunction with CIDP but has been associated with neuroendocrine disorders including postural orthostatic tachycardia syndrome (POTS), adrenal insufficiency, and autonomic neuropathy [10]. In our patient, the destruction of peripheral nerves may have led to inadequate peripheral vasoconstriction and the observed preload failure. Therefore, preload failure may be a diagnosis to consider when approaching a patient with unexplained dyspnea or postural symptoms and known small fiber neuropathy.

It is interesting that our patient presented with extensive thromboembolism two months prior to developing any neurological symptoms. However, even in the absence of clinical weakness patients with sensory predominant CIDP can demonstrate significant motor conduction slowing on nerve conduction studies [12]. This indicates that inflammation and destruction of peripheral nerves can occur in the absence of clinical symptoms. The clinically silent peripheral nerve damage and inflammation likely led to hypercoagulability and venous stasis, possibly leading to thromboembolism formation in this patient. Extensive VTE may therefore represent a unique early presenting sign in atypical forms of CIDP.

An increased risk of VTE has been reported in autoimmune neurological disorders such as multiple sclerosis and myasthenia gravis, but to our knowledge, no cases have previously been reported of VTE associated with CIDP [6]. It is imperative to determine whether CIDP patients are at increased risk because patients often have decreased mobility and are on therapies such as IVIG which may further increase thromboembolic risk.

However, our patient had not received any therapies or experienced significant mobility impairment prior to presenting with VTE, indicating that underlying systemic inflammation and peripheral denervation leading to venous stasis were the most likely potential mechanisms in this patient. Our case suggests an association between VTE and CIDP. Further studies will be required to determine whether CIDP patients are truly at increased risk for VTE.

## Conclusions

To our knowledge, no cases have previously been reported of VTE associated with CIDP. It is imperative to determine whether CIDP patients are at increased risk because patients often have decreased mobility which can further increase thromboembolic risk. However, our patient had not experienced significant mobility impairment prior to presenting with VTE, indicating that underlying systemic inflammation and peripheral denervation leading to venous stasis were the most likely potential mechanisms. Our case suggests an association between VTE and CIDP. Further studies are required to determine whether CIDP patients are truly at increased risk for VTE.
